# Action Observation Therapy in the Subacute Phase Promotes Dexterity Recovery in Right-Hemisphere Stroke Patients

**DOI:** 10.1155/2014/457538

**Published:** 2014-05-22

**Authors:** Patrizio Sale, Maria Gabriella Ceravolo, Marco Franceschini

**Affiliations:** ^1^Department of Neurorehabilitation, IRCCS San Raffaele Pisana, Via Della Pisana 235, 00163 Rome, Italy; ^2^Department of Experimental and Clinical Medicine, Polytechnic University of Marche, 60121 Ancona, Italy

## Abstract

The clinical impact of action observation (AO) on upper limb functional recovery in subacute stroke patients is recent evidence. We sought to test the hypothesis that training everyday life activities through AO coupled with task execution might activate the left hemisphere different from the right one. Sixty-seven first-ever ischemic stroke subjects were randomly assigned to receive upper limb training coupled with AO tasks or standard rehabilitation. The groups were matched by age and gender, Bamford category, and interval from stroke and lesion side. Fugl-Meyer (FM) and Box and Block Test (BBT) were used to measure hand function recovery at the end (T1) and 4-5 months after the treatment (T2). At T1, FM was increased by 31% (±26%), of maximum achievable recovery, whereas BBT was increased by 17% (±18%); at T2, FM had reached 43% (±45%) of maximum recovery, while BBT had reached 25% (±22%). Combining the effects of treatment to those of lesion side revealed significantly higher gains, in both FM and BBT scores, in left hemiparetic subjects when exposed to AO as compared to standard rehabilitation alone (*P* < .01). The findings lead to recommend the use of AO in addition to motor training in left hemiparetic patients.

## 1. Introduction

The most common and disabling motor deficit following stroke is the loss of upper limb function [1]. Functional recovery is known to be influenced by the size, type, and site of brain damage [2], as well as by the quality and intensity of the rehabilitation intervention. The current views on rehabilitation effectiveness advise to pursue the relearning of basic skills concerned with activities of daily living (ADL) and to practice ADL in an intensive manner in order to optimize the upper limb function [3].

Over the last few years, several approaches have been tested with respect to their efficacy at promoting hand dexterity recovery after stroke. Among them, task-oriented therapy, robot-assisted rehabilitation, and action observation were paid the greatest attention [4–7].

Action observation (AO) is defined as a dynamic state during which an observer can understand what other people are doing by simulating the actions and the outcomes that are likely to follow from the observed motor act [8]. In particular, the systematic observation of daily actions followed by their imitation represents a novel rehabilitation approach; AO exploits a well-known neurophysiological mechanism by which the brain matches an observed action to its motor counterpart [9]. This phenomenon is supposed to occur via the activation of the mirror neuron system (involving the inferior parietal lobule, the premotor cortex, and the superior frontal gyrus) [10]. Fadiga et al. [11] suggested that observation of action has a direct influence on primary motor cortex and muscle activity, thus supporting the idea that observation can prime movement execution by activating common neural processes. A research conducted by Buccino et al. [12] revealed that the mirror neuron system is especially active during the observation of actions which are part of the motor repertoire of the observer. In studies where AO was applied as a tool for promoting motor relearning, stroke patients were asked to observe everyday life actions (i.e., actions of high ecological value), of which they had motor competence and experience. The hypothesis that such aspects of the observed actions could trigger brain areas belonging to the mirror neuron system was strengthened by the finding that the motor improvement observed in patients undergoing an AO treatment, compared to controls undergoing standard rehabilitation, paralleled an increased activation in a network comprising bilateral ventral premotor and inferior parietal areas (supposedly containing the mirror neuron system) plus bilateral superior temporal gyrus, supplementary motor area (SMA), and contralateral supramarginal gyrus [13].

Gatti et al. showed that AO is better than motor imagery as a strategy for learning a novel complex motor task, at least in the early phase of motor learning, thus emphasizing its role in neurorehabilitation [14]. In a recent paper, we showed the clinical impact induced by 4 weeks of structured AO rehabilitation treatment at enhancing the upper limb functional recovery in subacute stroke patients. In particular, we demonstrated a persistently higher improvement in the Box and Block Test scores in the experimental group as compared to the controls [5].

In the present research, we combined AO with the direct effects of action execution to promote dexterity recovery in subacute stroke patients, with moderate to severe upper limb paresis. Based on the finding that the mirror neuron system is mostly activated by the observation of tasks of whom the observer has motor experience, we sought to test the hypothesis that training everyday life activities through AO coupled with task execution might activate the left hemisphere (i.e., the hemisphere contralateral to the dominant hand) to a different extent from the right hemisphere (i.e., the hemisphere contralateral to the nondominant hand).

Therefore, we designed a trial where right-handed people, surviving their first-ever ischemic stroke, were randomly assigned to receive either AO coupled with action execution or standard rehabilitation for 4 weeks.

## 2. Material and Methods

This is a randomized controlled observer-blind trial aimed at discriminating the effectiveness of AO, in left versus right hemiparetic subjects, receiving AO as an add-on treatment to the standard physical therapy in the early phase of stroke onset.

**Figure 1:**
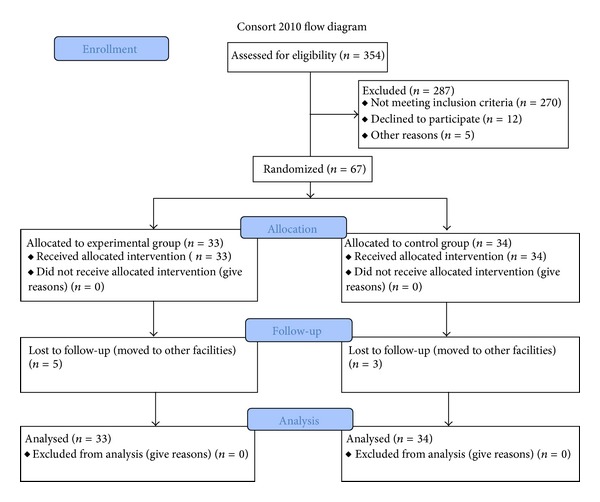
Consort 2010 flow diagram.

Eligible patients had moderate to severe upper limb paresis, following their first-ever ischemic stroke. According to our previous study protocol [5], we enrolled patients 30 days (±7) after the event (Figure 1). All patients were right handed prior to stroke and exhibited unilateral brain lesions.

The following exclusion criteria were identified: (1) posterior circulation infarction; (2) subarachnoid hemorrhage; (3) severe forms of neglect and anosognosia; (4) impaired comprehension or dementia; (5) history of endogenous depression or serious psychiatric disorders; (6) severe visual deficits; (7) bilateral motor impairment; (8) severe sensory deficits in the paretic upper limb; (9) refusal or inability to provide informed consent; and (10) other concomitant severe medical problems.

Diagnosis was confirmed by means of a CT scan and/or an MRI. The baseline assessment included the Canadian Neurological Scale (CNS), the Mini-Mental State Examination (MMSE), the Bell Barrage Test, and the ideomotor apraxia test (Spinnler-Rognoni).

Furthermore, the following functional evaluations were performed at the beginning (T0), at the end of the 4-week treatment period (T1), and 4-5 months from treatment conclusion (T2): the Fugl-Meyer Test (FM), with respect to the upper limb items [15], and the Box and Block Test (BBT) [16].

The Fugl-Meyer assessment is one of the most widely used quantitative measures of motor impairment. It has been applied in both the clinical and research setting to evaluate recovery in poststroke hemiplegic patients. Items are scored on a 3-point ordinal scale (0 = cannot perform; 1 = performs partially; 2 = performs fully). The five domains assessed include motor function (upper limb maximum score = 66; lower limb maximum score = 34), sensory function (maximum score = 24), balance (maximum score = 14), joint range of motion (maximum score = 44), and joint pain (maximum score = 44). Subscales can be administered without using the full test.

The BBT was devised to assess unilateral gross manual dexterity in stroke subjects. It requests patients to seat at a table, facing a rectangular box that is divided into two square compartments of equal dimension by means of a partition: one of the two compartments contains one hundred and fifty, 2.5 cm, colored, wooden cubes. The individual is instructed to move as many blocks as possible, one at a time, from one compartment to the other for a period of 60 seconds. The final score is computed by counting the number of blocks moved during the one-minute trial period. Healthy adults aged 20 and up have been found to move around 75 cubes ±9,1, within one minute, without any significant differences between the dominant and nondominant hand [16]. The interrater reliability and validity of FM and BBT are excellent [17].

All assessments were performed by a trained occupational therapist (OT) who was not aware of the research aims and treatment content. The local ethical committee approved the study. All patients gave informed consent to the investigation.

### 2.1. Subjects

The studied sample was made of 67 subjects (26 women), aged 66.5 ± 12.7 years (range: 28–87); the interval from stroke was 29.6 ± 4.5 days (range: 23–37); the left hemisphere was involved in 30 cases. Subjects were moderately disabled, their average Barthel index being 43.1/100 ± 20.6; the upper limb function (on the paretic side) was severely impaired as measured by a mean BBT of 8.9 ± 11.9 on the paretic side, compared to 69.1 ± 8.2 on the healthy side.

The subjects were randomly assigned either to the experimental treatment (EG, 33 cases) or to the control treatment (CG, 34 cases).

### 2.2. Random Group Allocation

The random allocation to treatment was concealed and based upon a custom computerized system, using dedicated software. Each participating center was provided with client software through which the local participant could ask the server, for any eligible subject, for the group allocation by simply entering his/her age, gender, and brain lesion side. The server could be solely accessed through the client software and an HTTPS Internet protocol. In order to allow for a balanced subject allocation into EG and CG groups, the Lehmer algorithm was applied. Therapists were randomly assigned to patients within each group, by using the same procedure.

All subjects underwent in-patient rehabilitation consisting of at least 3 hours/day of physiotherapy (60-minute practice of trunk control and standing, gait and balance training, and breathing exercise), occupational therapy (60-minute training of wheelchair locomotion and practice of activities of daily living), and speech and swallow therapy (60-minute practice of activities to enhance speech, articulation, fluency, and safe swallowing). No differences were admitted between right and left hemiparetic subjects with respect to the daily amount of formal upper limb training.

In addition to standard rehabilitation, eligible patients received two 15-minute daily sessions, 5 days/week, for 4 consecutive weeks, of either experimental (EG) or control treatment (CG), according to the random allocation outcome.

### 2.3. Experimental Treatment

Every day, before starting physical training, EG patients were asked to carefully watch footages showing 20 different daily routine tasks (actions) carried out with the upper limb [6]. The patient was presented only one task per day, starting from the easiest and ending with the most complex action throughout 20 sessions, the whole treatment period lasting 4 weeks (5 sessions/week). Each action consisted of three different meaningful motor sequences displayed in order of ascending difficulty and lasting 3 minutes each. Tasks were based on some relevant ADLs such as drinking from a glass, combing hair, opening a box, eating an apple, and more, all actions being object- and goal-directed. For example, take and drink a cup of coffee was divided into 3 acts: (1) reach and grasp the handle of the cup with the affected arm and return to the starting point; (2) reach and grasp the handle of the cup with the affected arm; rise the cup towards the mouth; return to the starting point; (3) reach and grasp the handle of the cup with the affected arm; rise the cup towards the mouth and drink; then, return to the starting point. There were unimanual and bimanual tasks. Unimanual tasks required the use of only the affected limb. The actions were observed from a first-person perspective. Actors in the videos were young nondisabled people, either men or women, different from video to video. During each daily session, the patient had to watch the video under OT supervision. In particular, subjects were asked to carefully observe the video, in order to prepare to imitate the presented action, whereas the OT consistently held high the patient's attention with verbal feedback. At the end of each sequence, the OT prompted the patient to perform the same movement over a time period of 2 minutes, providing help when needed. The patients were asked to perform the observed action with their paretic upper limb at their best convenience, as many times as they could. They received verbal instructions by the OT as follows: “slowly put the hand of your affected arm to the top of your head. You may use your unaffected arm to help guide if needed,” or “extend your affected arm to the wall in front of you. You may use your unaffected arm to help guide if needed.” The OT judged whether patients could accomplish the task themselves or should be assisted in the task of imitating the observed action. In the last case, the OT provided patients with physical help (limb support or passive mobilization) to help them perform the action. No interaction with object was allowed; patients had only to imitate the motor sequence they had observed. No movements in free space or manipulation were requested. Each session had to last about 15 minutes (3-minute sequence observation and 2-minute action performance for 3 motor sequences) and was repeated twice per day, in two separate sessions, at least 60 minutes apart; during the interval, the patient was requested to rest.

### 2.4. Control Treatment

Different from the experimental treatment, a “sham” action observation was used for CG patients. Subjects were shown 5 static images displaying objects, without any animal or human being, for 3 minutes [6]. The displayed objects were not manipulable and consisted of pictures of buildings, trees, cruise ships, mountains, beach umbrellas, beds, and tables. A cognitive task was required in order to keep the patient's attention at high concentration: for a 3-minute sequence, images were separately displayed, each for 30 seconds, and then overlapped all together during the last 30 seconds, as an intrusive image (interloper) that the patient was asked to identify so that his attention span could be checked in real time by the OT. For example, the images regarded seascapes, but, inside the sequence, there was an image with mountains. The subject had to identify the image with mountains. Subjects were then asked to perform limb movements (at their best convenience) for 2 minutes according to a standard sequence, simulating those performed by the EG, in what refers to shoulder and elbow joint mobilization. They received verbal instruction by the OT such as the following: “starting with your elbow flexed at an angle of 90° and your shoulder adducted, please abduct your shoulder and extend your elbow as far as you can. You may use your unaffected arm to help guide if need.” They did never interact with objects nor did they see the OT perform the movement. Movement was actively performed to the best of each subject's ability. As for EG, OT provided patients with physical help (limb support or passive mobilization) to help them accomplish the motor sequence. For each session, 3 different 3-minute sequences were displayed (each including 5 new images), thus leading to a 15-minute total duration of the rehabilitation session.

Both EG and CG received two treatment sessions/day at 60-minute interval apart. Every missed session was retrieved. Subjects who did not retrieve sessions and interrupted treatment for more than 5 consecutive days were excluded from the study.

### 2.5. Statistical Methods

The BBT was the primary outcome measure applied. It was chosen due to its validity and reliability as a dexterity measure in poststroke hemiplegic patients, whereas FM was deemed to assess upper limb gross motor function. Given the multiple endpoints measured in the study, the sample size was calculated according to the BBT, that is, to the parameter expected to benefit the most from the experimental treatment. We used the unpaired *t*-test to assess the homogeneity of the 2 groups at baseline for age, interval from stroke, and primary outcome measures. Moreover, in order to take into account both within-group and between-group changes at each time point, the “T1−T0” and “T2−T0” differences in FM and BBT scores were analyzed in a 2 × 2 repeated-measures analysis of variance (ANOVA); post hoc between-group comparisons were performed using the Mann-Whitney *U* test. In order to adjust the assessment of function improvement by score severity at entry, we applied a rehabilitation “effectiveness index,” which was computed for each outcome measure, at each time point, as follows: (score T f-up-score T0.)/(max. achievable score-score T0). The formula describes observed improvements as percentages of maximum achievable gains, thus balancing the observed score changes across subjects showing different neurological impairment at baseline, with an effort towards emphasizing the achievement of optimal scores [18]. The maximum achievable score for BBT was set, for each patient, upon the healthy upper limb performance.

## 3. Results

The distribution of the patients by age, gender, and main clinical characteristics, at baseline, did not significantly differ between the EG and the CG. All subjects completed the 4-week treatment period and fulfilled the assessment protocol at T1. Subsequently, five patients from EG and 3 from CG moved to a different rehabilitation facility and declined the invitation to the follow-up visit.

The assessment performed at both T1 and T2 showed a significant improvement in arm function, in the whole sample. The observed changes (computed as percentages of the maximum recovery potential) were as follows: 31% (±26%) for the Fugl-Meyer at T1, 43% (±45%) for the Fugl-Meyer at T2, 17% (±18%) for the Box and Block Test at T1, and 25% (±22%) for the Box and Block Test at T2. No effects of age, gender, and stroke aetiology on upper limb function recovery were ascertained. The comparison between groups revealed a significantly higher gain for the EG than the CG, with respect to functional measures taken at both T1 and T2. An interaction analysis combining the effects of treatment to those of lesion side revealed that left, though not right, hemiparetic subjects achieved significantly greater benefits, in both FM and BBT scores, when exposed to AO, compared to standard rehabilitation alone (Table 1).

## 4. Discussion

This study shows that action observation can stimulate and enhance the beneficial effects of motor training in left hemiparetic patients undergoing intensive rehabilitation in the subacute phase of ischemic stroke.

In agreement with previous studies [4–6, 19], our data suggests that observation of action, with the intention to imitate movements, can increase the excitability of the brain motor areas and, in doing so, can stimulate the recovery of motor control. Moreover, in addition to what has been already described by others, we hypothesized and observed that action observation, coupled with action execution, induces a higher improvement in right hemispheric compared to left hemispheric strokes.

It is fair to acknowledge that we did not carry out any electrophysiological or functional imaging studies to investigate the neural correlates of our clinical findings. The greater susceptibility of left, compared to right, hemiparetic strokes, to achieve functional benefits from a rehabilitation treatment implementing AO, cannot be easily explained. Recently, some researchers showed that the perception of tools, though not of other objects, activates the left premotor and somatosensory cortex, representing object affordances [20]. According to these authors, viewing tools automatically activates mental representations associated with their manipulation, independent of the kind of object and the type of grip (unimanual or bimanual). The left premotor cortex has been found to be involved with any kind of object and grip, as early as 200 milliseconds after stimulus presentation, thus supporting the hypothesis of a left hemisphere asymmetry in the neural representation of grasping, within this region. It may be hypothesized that right-handed people develop an asymmetrical representation of motor skills leading to a greater involvement of the left hemisphere during the observation of everyday life actions. Hamzei et al. recently proposed a connectivity model where the illusion of bimanual hand movement during mirror training (MTr) promoted functional coupling between each premotor region and the supplementary motor area (SMA) ipsilateral to the untrained hand, which in turn showed an increased functional interaction with the ipsilateral sensory motor cortex (SMC) [21]. More specifically, they proved that right hand training, in healthy subjects, led to performance improvement of the untrained left hand and that this finding was made possible by the involvement of the left SMA. They did not test their hypothesis in the reversed setup (i.e., trying to increase right hand performance via left hand training); therefore, they cannot exclude the assumption that left hemisphere is especially activated, during action observation, this being the key factor that prompts the achievement of motor practice effects, when movement is just observed. The emphasis on the role played by the left hemisphere networks in AO tasks, as can be inferred from the findings by Hamzei et al. [21], could provide a neurophysiological basis to our empirical findings of a selective improvement of left hemiparetic stroke subjects undergoing motor training coupled with AO; in fact, it could be hypothesized that daily tasks are especially represented in the left hemisphere of right-handed people and that the activation of the mirror neuron system of this side (which is spared in left hemiparetic subjects) is a key factor to the increase of activity of cortical motor areas. Of course, this hypothesis needs to be confirmed by the reverse finding of a greater benefit achieved through AO in right hemiparetic, left-handed subjects.

*Study Limitations*. The interpretation of group differences is only based on clinical measures and this methodological choice represents the main limitation of this research. Future studies combining electrophysiological recording or functional neuroimaging with data acquired using experimental psychology will hopefully provide a more comprehensive understanding of how action observation modulates the brain activity and the recovery of motor performance.

Nonetheless, the randomized and controlled design of this study, coupled with the blindness of assessments and the random allocation of therapists, supports the reliability of study findings. In fact, although our results concern subjects in the subacute phase of stroke, where spontaneous recovery is still expected, this possible source of bias was taken into account, by comparing the functional evolution of a moderate-severe upper limb paresis exposed either to an AO treatment or a “sham” treatment. Since the subjects in the EG and CG were matched with respect to clinical, functional, and demographic characteristics at baseline, spontaneous recovery might have equally concerned both groups, and, therefore, any further between-group difference should be regarded as treatment-related. The amount and kind of training received by each patient in the EG has possibly had its counterpart in the training received by a matched patient in the CG.

The choice of applying an effectiveness index, which describes observed improvements as percentages of maximum achievable gains, was aimed at adjusting motor recovery by score severity at entry, thus controlling for interindividual differences in the functional state before treatment. This kind of data processing may have helped to compensate the effects of the unavoidable differences in the amount and type of motor training undertaken by subjects showing different degrees of motor impairment at enrolment.

## 5. Conclusion

The results obtained in this research endorse the use of AO in addition to motor training in first-ever stroke survivors with left hemiparesis following right hemisphere damage. The positive findings obtained in subjects with moderate to severe upper limb paresis, the simplicity of treatment, and the lack of side effects strongly recommend extending the use of AO, in association with physiotherapy, to the early stage of stroke care. Larger future trials could be conducted enhancing the application of AO by the use of novel technology, in telerehabilitation protocols. Finally, further clinical trials exploiting fMRI to locate cortical reorganization following stroke, in a heterogeneous population, could challenge our hypothesis and define the real role of the left hemisphere network in brain-injured subjects.

## Figures and Tables

**Table 1:** Outcome measure score changes are displayed as percentages of recovery potential (effectiveness indices) at the end of the treatment (T1) and 4-5 months afterwards (T2).

	EG Mean ± SD (percentages of the maximum recovery potential)		CG Mean ± SD (percentages of the maximum recovery potential)		EG versus CG comparison *P* value
	T1	T2	T1	T2	
FM score change					
All subjects	40% ± 24% (33)		22% ± 25% (34)		**,003**
		56% + 32% (28)		30% ± 51% (31)	**,023**
RH subjects	42% ± 25% (15)		30% ± 29% (15)		n.s.
		64% ± 28% (12)		52% ± 39% (13)	n.s.
LH subjects	38% ± 24% (18 )		15% ± 18% (19)		**,003**
		50% ± 35% (16)		15% ± 54% (18)	**,029**
RH versus LH comparison *P* value	n.s.	**,027**	n.s.	**,042**	
BBT score change					
All subjects	23% ± 21% (33)		11% ± 14% (34)		**,012**
		31% ± 22% (28)		19% ± 21% (31)	**,031**
RH subjects	18% ± 21% (15)		15% ± 12% (15)		n.s.
		29% ± 2/% (12)		28% ± 18% (13)	n.s.
LH subjects	27% ± 20% (18)		8% ± 14% (19)		**,005**
		33% ± 23% (16)		13% ± 20% (18)	**,008**
RH versus LH comparison *P* value	n.s.	n.s.	n.s.	**,042**	

The results of between-group comparisons, by treatment and by lesion side, are reported. EG: experimental group; CG: control group; FM: Fugl-Meyer; BBT: Box and Block Test. RH: right hemiparetic; LH: left hemiparetic.
